# Clinical Features of Thiopurine-Induced Acute Pancreatitis: Comparison Between Patients With and Without Inflammatory Bowel Disease

**DOI:** 10.1093/crocol/otae072

**Published:** 2025-01-30

**Authors:** Tomofumi Oizumi, Yosuke Toya, Shunichi Yanai, Takayuki Matsumoto

**Affiliations:** Division of Gastroenterology and Hepatology, Department of Internal Medicine, School of Medicine, Iwate Medical University, Yahaba, Iwate, Japan; Division of Gastroenterology and Hepatology, Department of Internal Medicine, School of Medicine, Iwate Medical University, Yahaba, Iwate, Japan; Division of Gastroenterology and Hepatology, Department of Internal Medicine, School of Medicine, Iwate Medical University, Yahaba, Iwate, Japan; Division of Gastroenterology and Hepatology, Department of Internal Medicine, School of Medicine, Iwate Medical University, Yahaba, Iwate, Japan

**Keywords:** inflammatory bowel disease, thiopurine, acute pancreatitis, 5-aminosalicylic acid intolerance

## Abstract

**Background and Aims:**

Patients with inflammatory bowel disease (IBD) are at increased risk of developing acute pancreatitis (AP). Thiopurines (TP) are a well-known cause of AP. The aims of this study were to compare the incidence of AP and TP-induced AP (TIP) between patients with and without IBD under the use of TP and to assess for risk factors of TIP.

**Methods:**

We examined a retrospective cohort of 664 patients treated with TP from 2016 to 2021 at our institution. AP was defined as pancreatitis confirmed by symptoms, serum tests, and radiology, and TIP as AP occurring shortly after starting TP and improving after withdrawal. We compared the incidence of AP and TIP between patients with and without IBD and reviewed the clinical features of TIP patients in detail.

**Results:**

There were 366 IBD patients and 298 without IBD. IBD patients included 249 males (52.4%) with a median age of 39 years. Among them, 211 had ulcerative colitis (UC) and 155 had Crohn’s disease (CD). Azathioprine was administered to 560 patients, and 6-mercaptopurine to 104. AP occurred in 13 IBD patients but in none without IBD, with a significantly higher incidence in IBD patients (1.9% vs. 0%, *P* = .009). Seven of 13 patients with AP satisfied the criteria for TIP. Furthermore, 5 of the 7 TIP patients had a prior history of 5-aminosalicylic acid (5-ASA) intolerance.

**Conclusions:**

TIP may be a condition specific to IBD. IBD patients with 5-ASA intolerance are prone to TIP.

## Introduction

Inflammatory bowel disease (IBD), including ulcerative colitis (UC) and Crohn's disease (CD), is a chronic and refractory inflammatory disorder of the gastrointestinal tract.^1^ Thiopurines (TP) are immunomodulatory agents that play an important role in the treatment of IBD. TP are used for the treatment of patients with IBD, who are refractory or resistant to glucocorticoid (GC)^2^ and the maintenance of remission.^3–5^

Azathioprine (AZA) and 6-mercaptopurine (6-MP) are the TP that are currently used in the treatment of IBD. Myelosuppression and alopecia are the widely recognized, short-term side effects of those TP. Since a variant of nudie hydrolase 15 (*NUDT15*) (Arg139Cys) has been identified as the cause of these serious side effects,^6^ they have become avoided by *NUDT15* gene analysis prior to the start of TP. However, myelosuppression and alopecia may occur in a dose-dependent manner. There are other dose-independent side effects. Among those side effects, acute pancreatitis (AP) has been frequently reported in patients with IBD under TP use.^7^ It has also been reported that approximately 30% of patients with IBD have autoantibodies in the pancreas^8,9^ and that the incidence rate of AP in patients with IBD is higher than the overall incidence rate of AP in the Japanese population.^10,11^ Based on these observations, an association between pancreatic autoantibodies and the pathogenesis of TP-induced AP (TIP) has been suggested.^10^ However, the incidence rate of TIP in patients with and without IBD has not been fully investigated.

5-Aminosalicylic acid (5-ASA) is a key drug for the treatment of mild-to-moderate IBD. There have been many randomized controlled trials and systematic reviews on the efficacy and safety of 5-ASA for the induction and maintenance of remission in IBD.^12–15^ On the other hand, a certain proportion of patients receiving 5-ASA experience intolerance and/or allergy to the medication, which results in discontinuation. In addition, 5-ASA has been identified as a potential risk factor for AP.^16^ It has also been reported that 1.6% of patients with IBD treated by 5-ASA suffered from AP.^17^ However, the association between 5-ASA and TIP remains unclear.

In this retrospective cohort study, we aimed to elucidate the practical incidence of AP and TIP in subjects under the use of TP and to examine the contribution of 5-ASA in the pathogenesis of TIP.

## Patients/Materials and Methods

### Study Design

This was a retrospective, single-center cohort study. The study protocol was approved by the ethics committee of our tertiary care institution (Approval No. MH2022-076) and was conducted in accordance with the principles of the Declaration of Helsinki. Informed consent for study participation was obtained via an opt-out button on the website.

### Patients

**Figure 1.**
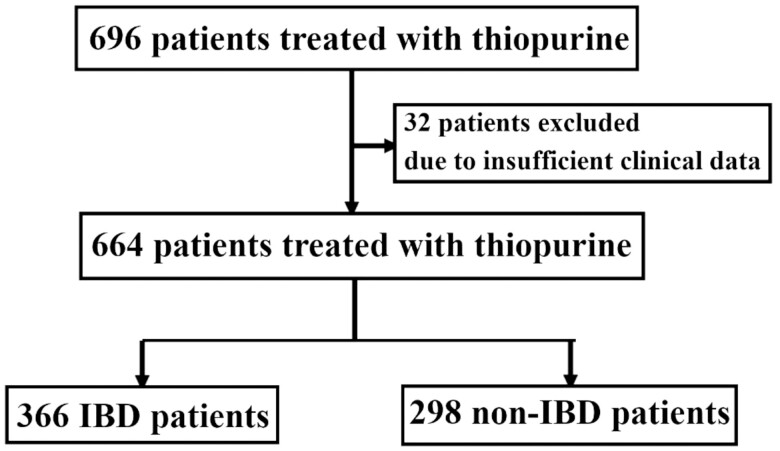
Flowchart of the study population. Between November 2016 and June 2021, 696 patients treated with thiopurines formulations were included in this study. Of these 696 patients, we excluded 32 patients who were not followed up within two months of the onset of treatment because of transfer or referral to other hospitals. Finally, a total of 664 patients were enrolled in this study.

We first recruited all the subjects who were prescribed AZA or 6MP of any dose at our institution during a period from November 2016 until June 2021. The initial search identified a total of 696 patients treated by TP. We then collected clinical information of each patient prior to the start until discontinuance of TP or the final visit. In patients treated with repeated periods of TP, we selected the first period as the target, because TIP usually occurs within 2 months after the start of TP.^9,18,19^ We also excluded 32 patients who were followed up for less than 2 months. As a consequence, we enrolled 664 patients in the study cohort. There were 366 patients with IBD and 298 patients without IBD (Figure 1).

### Definition of AP, TIP, and 5-Aminosalicylic Acid Intolerance

AP was defined as meeting at least 2 of the following 3 conditions: (1) acute onset of abdominal symptoms, (2) increase in serum amylase or lipase, (3) ultrasonographic or CT findings indicating obvious swelling of the pancreas with or without retroperitoneal fluid collection, and (4) exclusion of autoimmune pancreatitis based on blood biochemical tests and histological examination. TIP was defined as fulfilling the following conditions: (1) AP occurring within two months after the start of thiopurine therapy, (2) AP ameliorating upon withdrawal of the drug, and (3) exclusion of other causes of AP besides thiopurine therapy. The severity of AP was determined based on CT findings in accordance with the Japanese guidelines for acute pancreatitis.^20,21^

5-ASA intolerance was defined as high fever >38 °C together with diarrhea and/or abdominal pain occurring after the start of 5-ASA and ameliorating after its discontinuance without other treatment.^22^ In cases of 5-ASA intolerance, TP was initiated after symptom improvement.

### Statistical Analysis

Noncategorical variables were calculated as medians, range, and interquartile range (IQR) and were compared by the Mann–Whitney *U* test. Categorical variables were calculated as frequencies and percentages and compared by the Fisher exact test or the chi-squared test. *P* < .05 was considered to be statistically significant. SPSS software, version 27 Windows (SPSS Inc.) was used to perform statistical analysis.

## Results

### Incidence of AP and TIP

Table 1 summarizes comparisons of the demographics and clinical characteristics between patients with IBD and those without IBD. The median age at the time of TP administration was significantly younger and males were more predominant in patients with IBD than in those without IBD (39 vs 57 years, *P* < .0001; 52.4% vs 42.3%, *P* < .0001). Patients with IBD comprised 211 patients with UC (57.7%) and 155 patients (42.3%) with CD. Two hundred and ninety eight patients without IBD with TP use had the following underlying conditions; 64 patients (21.5%) with systemic lupus erythematosus, 46 (15.4%) with autoimmune hepatitis, 39 (13.0%) with acute lymphoblastic leukemia, 20 (6.7%) with microscopic polyangiitis, 13 (4.3%) with granulomatosis with polyangiitis, 7 (2.3%) with renal transplantation, 5 (1.6%) with autoimmune pancreatitis, and 104 (34.8%) with other underlying diseases. As to the species of TP, 6MP was more frequently prescribed in patients with IBD than in patients without IBD (22.7% vs 7.0%, respectively; *P* < .0001).

**Table 1.** Comparison of clinical characteristics and underlying disease in patients with and without IBD.

Variable	IBD (*n* = 366)		Non-IBD (*n* = 298)		*P* value
Age, years, median (range, IQR)	39	(16-92, 74)	57	(16-89, 29.8)	<.0001
Sex, *n* (%)					
Male	249	(52.4)	126	(42.3)	<.0001
Female	117	(47.6)	172	(57.7)	
Underlying conditions, *n* (%)					
Ulcerative colitis	211	(57.7)			
Crohn disease	155	(42.3)			
Systemic lupus erythematosus			64	(21.5)	
Autoimmune hepatitis			46	(15.4)	
Acute lymphoblastic leukemia			39	(13.0)	
Microscopic polyangiitis			20	(6.7)	
Granulomatosis with polyangiitis			13	(4.3)	
Post-renal transplantation			7	(2.3)	
Autoimmune pancreatitis			5	(1.6)	
Others			104	(34.8)	
Thiopurine, *n* (%)					
Azathioprine	283	(77.3)	277	(93.0)	<.0001
6-MP	83	(22.7)	21	(7.0)	
All acute pancreatitis, *n* (%)	13	(3.5)	0		.001
Thiopurine-induced AP	7	(1.9)	0		.019
Non-thiopurine-induced AP	6	(1.6)	0		.035
Gallstone pancreatitis	4	(1.2)	0		.068
Alcoholic pancreatitis	1	(0.2)	0		N/A
Pancreaticobiliary maljunction	1	(0.2)	0		N/A

Abbreviations: 6-MP, 6-mercaptopurine; AP, acute pancreatitis; IBD, inflammatory bowel disease; IQR, interquartile range.

In the cohort, AP occurred in 13 patients with IBD. In contrast, AP did not occur in any patients without IBD. Seven of 13 patients with AP fulfilled the criteria for TIP. It was thus calculated that the incidence of AP (3.5% vs 0%, *P* < .001) and TIP (1.9% vs 0%, *P* < .019) was significantly higher in patients with IBD than in patients without IBD. In patients with IBD who developed AP, the causes other than TIP included gallstone (4 patients), alcohol (a patient), and pancreaticobiliary maljunction (a patient).

### Clinical Features of AP and 5-ASA Intolerance

Table 2 shows the clinical characteristics of 13 patients with AP. In the TIP patients (*n* = 7), the median age of the patients was 33.0 years and 4 patients were male. Five patients had UC and 2 patients had CD. There were 2 current smokers among the TIP patients with UC. Six patients had wild-type NUDT15 while the remaining patient had heterozygous variant (Arg/Cys). All the patients were treated with AZA at a dose of 25 mg/day in 4 patients and 50 mg/day in 3 patients. The median (range) from the start of TP to the onset of AP was 18 days (13–28). The median number and the range of laboratory data were 270 (100–720) mL for eosinophil count, 216 (143–528) U/L for serum amylase, and 3.9 (0.1–16.4) mg/dL for serum C-reactive protein. The CT grade was 1 in all the patients who underwent CT. The median (range) from discontinuation of TP to improvement of abdominal pain was 6 days (4–8). Five of the 7 patients were found to be intolerant to 5-ASA. Symptoms of 5-ASA intolerance included fever in three patients, abdominal pain in 1 patient, and worsening diarrhea in 3 patients. In addition, 1 patient reported arthralgia, and another had hyperamylasemia. The median (range) from diagnosis of 5-ASA intolerance to the onset of TIP was 78 days (43–255).

**Table 2.** Clinical characteristics of patients with acute pancreatitis.

Age	Gender	Underlying conditions	smoking	NUDT15	Azathioprine (mg)	Duration to onset of AP (days)	Other causes of pancreatitis besides TP	Eosinophil count (/mL)	Amylase (U/L)	CRP (mg/dL)	CT grade	Duration to symptoms improvement (days)	5-ASA intolerance	Symptoms of 5-ASA intolerance				Duration from diagnosis of 5-ASA intolerance to the onset of TIP (days)
														fever	abdominal pain	diarrhea	others	
22	Female	UC	-	Arg/Arg	25	18	ー	250	528	16.4	1	6	Positive	+	+	+		212
33	Male	UC	+	Arg/Arg	50	19	ー	720	340	1.6	1	4	Positive	+	ー	ー	Arthralgia	43
26	Female	CD	ー	Arg/Cys	50	28	ー	100	143	3.9	1	5	Negative	ー	ー	ー		ー
40	Male	UC	ー	Arg/Arg	25	20	ー	360	187	10.3	1	6	Negative	ー	ー	ー		ー
56	Male	UC	+	Arg/Arg	50	13	ー	500	207	0.5	Not performed	7	Positive	+	ー	+		50
36	Male	CD	ー	Arg/Arg	25	14	ー	270	244	0.1	Not performed	8	Positive	ー	ー	ー	Hyperamylasemia	78
25	Female	UC	ー	Arg/Arg	25	17	ー	140	216	8.5	1	4	Positive	ー	ー	+		255
72	Male	UC	ー	Arg/Arg	75	38	Gallstones	70	1470	3.2	2	90	Negative	ー	ー	ー		ー
49	Female	CD	ー	Arg/Arg	50	42	Pancreaticobiliary maljunction	170	1720	3.3	2	35	Negative	ー	ー	ー		ー
70	Female	UC	ー	Arg/Arg	50	17	Gallstones	70	640	5.8	2	42	Negative	ー	ー	ー		ー
36	Male	CD	ー	Arg/Arg	50	32	Gallstones	110	842	10.3	1	28	Negative	ー	ー	ー		ー
72	Male	UC	ー	Arg/Arg	50	28	Alcoholic	140	324	6.2	2	25	Negative	ー	ー	ー		ー
38	Male	CD	ー	Arg/Arg	25	30	Gallstones	100	781	7.2	1	15	Negative	ー	ー	ー		ー

Abbreviations: 5-ASA, 5-aminosalicylic acid; Arg, arginine; CD, Crohn disease; CRP, C-reactive protein; Cys, cysteine; NUDT15, nudix hydrolase 15; TIP, thiopurine-induced acute pancreatitis; TP, thiopurine; UC, ulcerative colitis .

The clinical characteristics of the non-thiopurine-induced pancreatitis patients (*n* = 6) are also summarized in Table 2. The median age of the patients was 59.5 years and 4 patients were male. Three patients had UC and 3 patients had CD. There were no current smokers among the patients. All patients had wild-type NUDT15. Five patients were treated with AZA at a dose of 50 mg/day, another patient was under 25 mg/day, and the remaining patient under 75 mg/day. The median (range) from the start of TP to the onset of AP was 31 days (17–42). The median number and the range of laboratory data were 105 (70–170) mL for eosinophil count, 811.5 (324–1720) U/L for serum amylase, and 6 (3.2–10.3) mg/dL for serum C-reactive protein. The CT grade was 1 in two patients and 2 in 4 patients. The median (range) time duration from the start until improvement of symptoms was 31.5 days (15–90). No patients were intolerant to 5-ASA.

## Discussion

To elucidate the association between TP and AP in IBD, we compared the incidence rates of AP and TIP in 366 patients with IBD and 298 patients without IBD. The results revealed that the incidence of TIP was significantly higher in patients with IBD (1.9%) than in patients without IBD (0%). Furthermore, we found that 5 of 7 IBD patients with TIP had preceding intolerance to 5-ASA, suggesting a potential association between TIP and 5-ASA intolerance. To the best of our knowledge, this is the first report suggesting an involvement of 5-ASA intolerance in the development of TIP.

It has been well documented that patients with IBD are prone to the development of AP. It has also been known that there are various specific causes of AP in patients with IBD. The frequent causes are gallstones, autoimmune pancreatitis due to underlying IBD, and medications applied for the treatment of IBD. The major causative medications include glucocorticoids, 5-ASA, and TP.^23,24^ The clinical features of TIP have been reported to be a trend toward less severe pancreatitis.^24,25^ As has been reported in a previous study,^25^ our study showed that the duration of pancreatitis tended to be shorter in patients with TIP than in patients with non-thiopurine-induced pancreatitis (6 days vs 31.5 days, median).

The respective incidence rates of AP and TIP in our patients with IBD were 3.5% and 1.9%. These results were similar to those previously reported in the literature.^10,18^ Although the exact pathogenesis of TIP is unknown, previous reports have identified females, smoking, HLA class II region (rs2647087), and CD as risk factors for TIP.^24–26^ On the other hand, previous studies have reported conflicting results on the incidence of TIP in CD and UC. While several studies have reported a higher prevalence of TIP in patients with CD,^10,19,24,25^ others have not found an obvious difference in the incidence between the 2 diseases.^18,27^ Our results indicated a trend toward a higher incidence of TIP in patients with UC than in patients with CD. In addition, we found that 5 of the 7 patients who developed TIP had prior intolerance to 5-ASA. These observations suggest that the risk of TIP should be taken into consideration in UC patients who show intolerance to 5-ASA.

We did not encounter any non-IBD patients who had AP or TIP under the use of TP. A previous genome-wide association study (GWAS) of human leukocyte antigen (HLA) genetic regions found an association between TIP and the single nucleotide polymorphism (SNP) in rs2647087, which maps to the HLA-DQA1*02:01-HLA-DRB1*07:01 haplotype.^28^ The study showed that the rate of TIP in carriers of wild allele (C/C) or heterozygous variant (A/C) was higher than that in carries of homozygous variant, and as a consequence, the risk of TIP was 2.5 times higher in A/C carriers and 5 times higher in C/C carriers. However, data on the risk of TIP are not yet conclusive and therefore are not clinically applicable. Our observation suggests that the SNP in rs2647087 may be unrelated to TIP because there have not been any data indicating the association between rs2647087 in IBD.

It has been reported that approximately 10% of patients with UC have intolerance to 5-ASA, and, more importantly, the rate of intolerance has been increasing 3-to-4 folds during the latest 10 years.^22^ The increase is predominant in young and female UC patients. Furthermore, the drug-induced lymphocyte stimulation test (DLST) was positive in only 25% of UC patients intolerant to 5-ASA.^29^ In contrast, a recent GWAS has shown that a candidate SNP in rs144384547 in regions of the regulator of G-protein signaling 17 (*RGS17*) gene on chromosome 6 was associated with 5-ASA-induced fever and diarrhea in patients with IBD. However, the mechanism by which the SNP affects 5-ASA intolerance remains unknown.^17^ Based on these results, it can be suggested that 5-ASA intolerance is not a drug allergy, but it may be a side effect caused by multiple factors, including genetic factors.

Although 5-ASA intolerance may be associated with the development of TIP, the details of this relationship are unclear. In this study, the median (range) from diagnosis of 5-ASA intolerance to the onset of TIP was 78 days (43–255), and all patients diagnosed with 5-ASA intolerance discontinued medication. Considering that the half-life of 5-ASA is approximately 32 hours at most, it is unlikely that the pharmacological effects of 5-ASA remain in the body at the onset of TIP cases. This suggests that 5-ASA intolerance and TIP are unlikely to be caused by a simple interaction of the 2 drugs.

While the association of 5-ASA with TIP shown in our cohort may be accidental, the provisional mechanisms of the latter under the intolerance of the former need to be discussed. First, cross-reactivity in the immunological recognition of TP and 5-ASA by an IgE antibody is a candidate, which explains TIP under 5ASA intolerance.^30^ However, the cross-reactivity seems to be unlikely, because there are limited or null structural similarities between 5-ASA and TP. Second, drug sensitization, known as a process induced by the initial drug to allergic reactions to other drugs, maybe another and more appropriate explanation. On that occasion, the time duration until the resolution of drug sensitization varies from person to person. This is because several factors, including the intensity of the sensitization, the type of drug, and the immune response, affect resolution.^31^

The present study has several limitations. First, we failed to show any evidence indicating the association of 5ASA intolerance and TIP, because this was a retrospective and observational study. Second, the definition of 5-ASA intolerance has not yet been established. Additional prospective controlled studies with larger cohorts are warranted to confirm and clarify the relationship between 5-ASA intolerance and TIP in patients with IBD.

In conclusion, the incidence of TIP was significantly higher in patients with IBD than in patients without IBD. Moreover, more than half the patients with IBD who developed TIP had a history of 5-ASA intolerance prior to the administration of TP. A large prospective multicenter study is needed to validate our findings and clarify the relationship between 5-ASA intolerance and TIP in patients with IBD.

## Data Availability

The data that support the findings of this study are available from the corresponding author, corresponding author, upon reasonable request.
